# Risk Prediction and New Prophylaxis Strategies for Thromboembolism in Cancer

**DOI:** 10.3390/cancers13071556

**Published:** 2021-03-29

**Authors:** Patrizia Ferroni, Fiorella Guadagni, Mario Roselli

**Affiliations:** 1 Department of Human Sciences and Quality of Life Promotion, San Raffaele Roma Open University, 00166 Rome, Italy; fiorella.guadagni@sanraffaele.it; 2 InterInstitutional Multisciplinary Biobank (BioBIM), IRCCS San Raffaele Roma, 00166 Rome, Italy; 3 Department of Systems Medicine, Medical Oncology, University of Rome “Tor Vergata”, 00133 Rome, Italy; mario.roselli@uniroma2.it

Venous thromboembolism (VTE) is a compelling challenge across all phases of cancer care as it may result in treatment delays, impaired quality of life (QoL), and increased mortality [1]. This Special Issue of *Cancers* contains a series of articles presented by international leaders, focusing on the current clinical evidence supporting the standard of care and emerging therapeutic/prophylactic options for cancer-associated VTE during both active treatment and simultaneous/palliative care. Tailored approaches based on the use of individualized factors to stratify the thrombotic/bleeding risk in each individual patient are also discussed.

The increased risk of VTE in cancer is typically related to patient [2,3], tumor [2,4], and/or treatment [5,6,7,8,9], which may all cause a disruption of each component of Virchow’s triad, altering the haemostatic mechanisms that balance thrombosis and clot lysis and, thus, increasing hypercoagulability. Here, Nasser and colleagues propose an interesting stratification of cancer-related hypercoagulability into two main types: Type I hypercoagulability (resulting from the degradation of endogenous heparin by tumor-secreted heparanase) and Type II hypercoagulability (including all the other etiologies) [10]. Heparanase, indeed, is capable of degrading heparin and low-molecular-weight heparin (LMWH), possibly resulting in neutralization of the anticoagulant properties of these molecules [9]. Interestingly, heparanase was found to be highly expressed in pancreatic, gastric, and lung cancers, which are all correlated with a higher risk of thrombosis compared to other tumor types [10]. Accordingly, Nasser et al. speculate that developing alternative non-invasive methods to deliver heparin—or to mobilize endogenous heparin from its reservoirs (i.e., platelets)—could make this medication more appealing to treat cancer-associated thrombosis [10].

Other tumor-related factors can concur to represent additional triggers of VTE in cancer patients. In this context, the advent of tumor genomic profiling has strongly contributed not only to a deeper comprehension of cancer biology, but also to the discovery of potential VTE risk genomic factors. The subject is addressed in the review by Leiva and colleagues discussing the potential mechanisms by which the tumor mutational status may influence thrombogenesis [11]. Molecular aberrations involving various targetable driver mutations may, in fact, impact thrombotic risk in many tumor types, possibly through a disregulation of tumor tissue factor (TF) expression. This is the case of mutated *KRAS* in colorectal and lung cancer and *IDH1* in brain cancer patients, the former being positively associated with TF upregulation, the latter being associated with hypermethylation of the F3 promoter of the TF gene leading to decreased TF expression and a decreased risk of VTE [11]. Other tumor mutations that have been involved in the prothrombotic state in carcinoma patients include *ALK*, *ROS1*, and *JAK2*, all participating in downstream signaling of inflammatory cytokines [11], while the burden of breast cancer mutational events, using a next-generation sequencing approach, is currently the focus of an ongoing trial [12]. Knowledge that a patient may be at an increased thrombotic risk due to the underlying tumor genotype is another piece of information that the treating clinician can consider when determining VTE risk stratification and may prove to be a significant advancement in the prevention of cancer-associated thrombosis [11].

While many factors contribute to increase the individual thrombotic risk, some co-morbidities and/or chemotherapy-related side effects, such as renal or hepatic insufficiency and thrombocytopenia, can affect the efficacy and safety of anticoagulation, emphasizing the need for a careful evaluation of the risk/benefit assessment of anticoagulant prophylaxis. Hence, the availability of tailored approaches—based on the use of individualized factors to stratify the thrombotic/bleeding risk in each individual patient—undoubtedly represents a significant advance in the prevention of cancer-associated VTE. Several clinical decision models have been developed to guide the oncologist in thromboembolic risk assessment and targeted prophylaxis. The article by Mulder et al. addresses some of the controversies stemming from the translation of the guideline recommendations into clinical practice, discussing the performance of available risk assessment scores, and summarizing the findings of recent trials [13]. From their analysis (performed in light of the most recent prophylactic options), it emerges that the development of an efficient pan-cancer VTE prediction score—as those currently available—is probably not feasible, given the large heterogeneity in tumor biology, cancer treatment, and thromboembolic risk across cancer types [13]. In the authors’ opinion, with which we agree, prediction scores should possibly be developed for specific cancer types to help effectively individualize strategies for primary thromboprophylaxis in cancer patients.

The state-of-the-art current guidelines on thromboprophylaxis in cancer patients is addressed in the reviews by Labianca et al. [14] and Rossel et al. [15], summarizing the latest evidence on VTE prophylaxis and treatment in patients with cancer. From their analyses, it emerges that the use of VTE prophylaxis is currently recommended in cancer patients following surgery, or if admitted to hospital for an acute medical condition, but large-scale thromboprophylaxis prescription in ambulatory cancer patients is not advised. Based on the latest recommendations, prophylaxis should always be practiced in high-risk patients with multiple myeloma and in therapy with lenalidomide or thalidomide, unless there are specific clinical contraindications. On the other hand, primary prophylaxis is recommended in outpatients receiving systemic anticancer therapy at an intermediate- to high-risk of VTE—identified by cancer type (i.e., pancreatic) or by a validated risk assessment model (i.e., a Khorana score ≥2)—and not at a high risk of bleeding. Thus, patient selection remains the main challenge and improvement of existing VTE risk models, or construction of alternative risk assessment models are needed in order to ameliorate the risk stratification of cancer patients [14,15].

One of the most important novel developments that can be found in the latest recommendations by expert societies is the endorsement of the use of edoxaban and rivaroxaban for VTE treatment/prophylaxis in cancer patients [14,15]. Direct oral anticoagulants (DOACs) represent an interesting option because of their oral administration and lower costs compared to low-molecular-weight heparin (LMWH). Large scale thromboprophylaxis prescription in ambulatory cancer patients is still not advised. However, based on evidence from the AVERT and CASSINI trials, it is now recommended that patients with cancers at very high VTE risk (e.g., pancreas) may be offered thromboprophylaxis with DOACs, whereas caution is needed in patients with GI and genitourinary cancers.

The use of DOACs in VTE treatment and primary prevention in cancer patients is the focus of the review by Wojtukiewicz and coworkers [16]. LMWH has been the recognized standard drug for more than a decade, until recent published results of large randomized clinical trials have confirmed that DOACs may represent a reasonable alternative to LMWH in cancer patients—both in terms of efficacy and safety—and a valuable step forward in the treatment and prevention of cancer-related thrombosis [16]. As stated above, DOACs are an alternative to LMWH in the recommendations of expert societies [14,15,16] both in the treatment of cancer-associated thrombosis and in VTE primary prevention in high-risk patients [15,16]. Limitations of DOACs are also discussed, including the increased risk of major bleeding, interaction with other drugs, unknown or inappropriate pharmacokinetics in patients with large deviations from normal body weight and in patients with impaired renal function, corroborating the need for careful patient selection [16].

Our digression on the topic of VTE risk stratification and antithrombotic prophylaxis continues with some examples focusing on some high-risk tumor types. The first of these reviews is that by Farge and colleagues [17] who address the issue of clinical practice guidelines on primary thromboprophylaxis in pancreatic cancer (PC) patients [17]. PC is a malignancy with the highest mortality rate of any solid cancer and with the highest rate of VTE. In their article, Farge et al. interestingly point out that despite the fact that Grade 1B evidence has been long since available and thromboprophylaxis is generally recommended in clinical practice guidelines, this remains largely underused in PC patients [17]. Clinical tools could be used to assist clinicians in optimizing treatment in daily clinical practice. However, in the Khorana score, all PC patients have a sum score ≥2 and should, therefore, be considered for prophylaxis. Other models, including those reviewed by Mulder et al. [13], have not been externally validated in ambulatory PC patients. The authors conclude that, in the absence of clear evidence to favor either LMWH or DOACs, a “discussion with the patient about the relative benefits and risks, drug cost, duration and tolerance of prophylaxis is warranted before prescribing thromboprophylaxis in PC ambulatory patients” [17].

Fotiou et al. [18] and Hohaus et al. [19] further address the issue of VTE risk stratification and thromboprophylaxis in hematological malignancies. The first article is focused on the need for the development of more accurate risk assessment tools and measures of thrombosis prevention in multiple myeloma (MM) patients [18]. As argued by Fotiou and colleagues, optimum risk stratification and effective thromboprophylaxis can only be achieved through the development of an MM-specific risk score that can successfully capture all aspects of the heterogeneous prothrombotic environment that exists in MM patients to accurately stratify VTE risk and guide thromboprophylaxis [18]. As proposed by the authors, a risk assessment tool including clinical- and treatment-specific risk factors in combination with disease-specific coagulation biomarkers could allow the successful use of the right agent for the right patient and for the sufficient amount of time. An ideal/future algorithm for VTE risk prediction—based on the IMPEDE risk score—using information from current expert society guidelines, data from randomized controlled trials, emerging data on DOACs, retrospective MM VTE risk prediction clinical scores, and clinical experience is also proposed [18]. Similar considerations are raised by Hohaus and co-workers, who review the epidemiology of VTE, its prevalence, and tumor-related factors in lymphoma patients [19]. In agreement with the opinion by Mulder et al. [13], the authors suggest that the pan-cancer Khorana score (developed for patients with solid tumors) is not fitted to capture the disease-specific characteristics associated with VTE risk in lymphoma [19]. Given the absence of a validated risk score, no evidence-based recommendation for VTE prophylaxis in ambulatory patients undergoing anti-neoplastic treatment can be given at present and individual evaluation of the risk–benefit ratio is the current strategy [19].

Finally, Riondino et al. [20] and Zabrocka et al. [21] address the issue of venous thromboembolism in particular settings of cancer care: the transition from active to palliative care, and end-of-life care, respectively. Based on the most recent NICE (National Institute for Health and Care Excellence) guidelines, thromboprophylaxis should be considered for patients receiving palliative care, always taking into account several factors, including risk of bleeding, estimated life expectancy, and the views of the patient and their caregivers. Additionally, VTE prophylaxis should be reviewed daily and should not be offered in the last days of life. Other factors to be considered include the lack of palliative benefits or any unreasonable burden of thromboprophylaxis (e.g., painful injections or frequent monitoring with phlebotomy). Nonetheless, from the analysis by Riondino and colleagues, it emerges that the prevalence of VTE among palliative care unit patients is significant (35 to 50%), and its occurrence is perceived as a physically and emotionally distressing phenomenon that overlaps with the underlying malignancy and strongly decreases QoL [20]. In end-of-life care, where the assurance of the best possible QoL should be the highest priority, VTE prophylaxis may eliminate the symptom burden and psychological distress related to thrombosis [21]. In light of the above, Riondino et al. emphasize that an early integration of VTE preventive strategies in a “simultaneous care program” might help overcoming the problem of deciding in favor or against thromboprophylaxis in the context of palliation [20]. However, specific decision-making tools are needed to avoid under-treatment, and since the continuum of care paradigm is in constant change, a major effort should be made in this area to achieve a broad consensus on how to manage VTE [20].

From the aforementioned data, it emerges that a large series of experimental and clinical data has given a tremendous impulse to disentangle the issue of VTE risk assessment in cancer, tracing new horizons for thromboprophylaxis in selected at-risk patients. A common need for new tools clearly emerges. As a consequence, clinical decision-making is rapidly moving from empiricism to customized healthcare and tailored therapy. Adjunctive clinical risk factors, biomolecular markers, and dynamic risk assessment could all ameliorate VTE prediction, while the introduction of novel computational analyses could help with gaining knowledge from available datasets to obtain accurate and precise personalized risk estimates.
